# Treating a Refractory Locally Advanced Carcinoma of the Cervix With Cone-Beam Computed Tomography-Based Adaptive External Beam Radiotherapy: A Case Report

**DOI:** 10.7759/cureus.70215

**Published:** 2024-09-25

**Authors:** Aaditya Prakash, Amitabh Kumar Upadhyay, Subhankar Show, Ajithkumar Mani

**Affiliations:** 1 Radiation Oncology, Tata Main Hospital, Jamshedpur, IND; 2 Medical Oncology, Tata Main Hospital, Jamshedpur, IND; 3 Radiation Oncology, Meherbai Tata Memorial Hospital, Jamshedpur, IND

**Keywords:** 3d-conformal radiation therapy, adaptive radiation therapy, cancer cervix, concomitant chemoradiation therapy, cone-beam computed tomography (cbct), hdr (high dose rate) brachytherapy, volumetric modulated arc therapy

## Abstract

Adaptive radiotherapy (ART) refers to methods that allow a radiation therapy plan to be adjusted based on images obtained during the treatment. Using cutting-edge imaging methods such as computed tomography (CT), magnetic resonance imaging (MRI), and positron emission tomography (PET), ART can adjust the treatment plan in response to observed changes in anatomy and even biology while the patient is receiving treatment. The backbone of ART is intensity-modulated RT (IMRT), which permits better sparing of normal critical organs while still delivering a uniform dose to target tumor volume. Volumetric modulated arc therapy (VMAT) is a more rapid form of IMRT with more conformity, which helps in treating patients in a shorter time. Different types of ART include individualized margins using an internal target volume (ITV) and offline and online methods. ITV uses the margin to appropriately cover the clinical target volume (CTV) based on matching CT scans to different extents of the radiological anatomy of the selected area. Offline adaptive strategies include scheduled replanning throughout the external beam radiotherapy (EBRT) course, depending on intra-fraction or inter-fraction changes. The online ART (oART) strategy takes into account changes in tumor volume and the daily anatomical variations of target volumes and organs at risk structures (OARS). As such, PTV margins have the potential to be reduced. Commercially available oART systems are predominantly MRI-guided, but more recent advances have seen the creation of a cone-beam CT (CBCT)-guided oART system. In this case of FIGO (International Federation of Gynaecology and Obstetrics) stage IIB squamous cell carcinoma of the uterine cervix, we used an offline ART approach to complete the initial part of the treatment, which included concurrent chemoradiation therapy with 50 Gy/25 Fr and weekly cisplatin for five weeks. However, in the final fraction of on-couch kilovoltage CBCT (kvCBCT), it appears that the tumor only partially responded, demonstrating its refractory nature to treatment. The patient then underwent a repeat planning contrast-enhanced CT (CECT) scan, which was fused with the initial planning CECT scan. It revealed that the tumor responded poorly, with only a slight decrease in size. With the OARS toxicity limit in mind, the patient was scheduled for an adapted volumetric modulated arc therapy (VMAT) boost of 8 Gy/4 Fr as a second-phase plan for the tumor. Subsequently, the patient was taken up for intra-cavitary brachytherapy (ICBT) after a one-week gap. She received brachytherapy with 9 Gy/session for two sessions as per institutional protocol on a weekly basis. On subsequent follow-up, the patient underwent a complete response clinico-radiologically, even after two years of follow-up. This case report shows the importance of adaptive radiotherapy in treating tumors with a high therapeutic ratio and less toxicity to OARS despite employing the less frequently used EBRT boost along with ICBT brachytherapy.

## Introduction

External beam radiotherapy (EBRT) and brachytherapy are the two most common forms of radiation therapy used to treat gynecologic tumors. When treating gynecological tumors with high-precision EBRT, such as intensity-modulated radiation therapy (IMRT), the motion of both the targets and surrounding normal structures is a matter of concern. The small bowel, bladder, and rectum are among the normal pelvic tissues that are organs at risk (OAR) for toxicity and are relatively spared with IMRT because they conform the radiation dose more closely to the target volume [1-3].

In India, the age-adjusted incidence of cervical cancer (30.7 per 100,000 women, 132,082 incident cases) is relatively high compared to all other types of cancer and higher than the South Central Asia region's average. The number of new cervical cancer cases in India is projected to increase to 226,084 by 2025 [4,5]. With such a large number of cases, it is critical to treat a large number of patients with conformal radiation therapy in order to increase survival and improve quality of life. Any organ motion in the context of such conformal treatment may result in geographical target misses and compromised outcomes.

Therefore, contemporary conformal radiotherapy with narrow margins and steep dose gradients, such as volumetric modulated arc therapy (VMAT) and IMRT, permits a decrease in the amount of radiation that is unwanted for healthy organs. VMAT is a more conformal and rapid form of IMRT. However, these techniques necessitate more accurate daily tumor imaging before every fraction and setup correction for the duration of the treatment. This is to avoid any target misses that may occur geographically.

Adaptive radiotherapy has emerged as a promising avenue to minimize toxicity to OAR while delivering ablative radiotherapy doses. Herein, we describe a case of carcinoma cervix in which, with the use of kilovoltage cone-beam CT (kvCBCT), the tumor response was assessed, and then an EBRT boost with the adaptive plan was delivered. Subsequently, brachytherapy was given, and in follow-up scans, the tumor was completely resolved without any long-term genitourinary (GU) or gastrointestinal (GI) toxicity [6-8].

## Case presentation

A 38-year-old premenopausal female without any comorbidities was evaluated for complaints of irregular bleeding per vagina since February 2022. Her general condition was good, with an average build. The Eastern Cooperative Oncology Group (ECOG) performance score was 1. On the per speculum examination, there was growth in the cervix region with upper vaginal involvement.

On per vaginal examination, there was growth of 5x5 cm, completely replacing the cervix and involving the upper third of the vagina with bilateral parametrium induration. The rectal mucosa was free. Bleeding was present on touch. A biopsy from the cervical lesion showed moderately differentiated squamous cell carcinoma.

**Figure 1:**
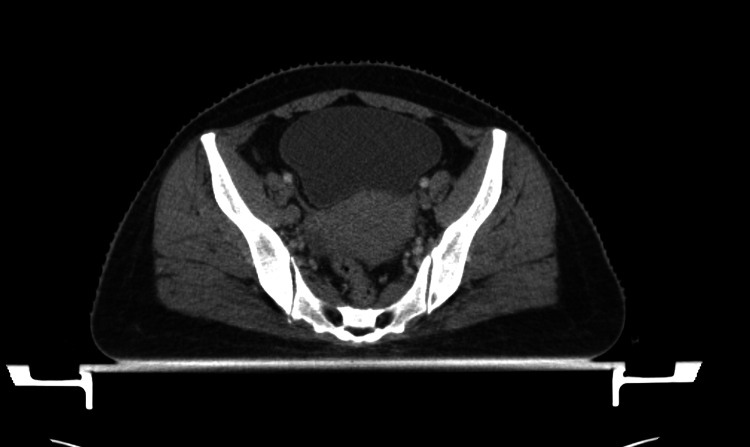
Baseline CECT of the whole abdomen (axial section) displaying sub-centimetric lymph nodes along with the main tumor. CECT: contrast-enhanced computed tomography

A contrast-enhanced CT (CECT) scan of the whole abdomen was suggestive of a 5.7x4.8x5.3 cm cervical mass lesion, predominantly involving the right parametrium and sparing pelvic wall. Involvement of the upper-third vagina is seen. The fat plane was maintained with the bladder and rectum. Sub-centimetric-size lymph nodes are observed in the bilateral obturator and iliac locations, suggestive of a reactive nature. The rest was normal (Figure 1).

The CECT of the thorax indicated the absence of lung metastasis. The patient was classified as International Federation of Gynaecology and Obstetrics (FIGO) stage IIB. She was scheduled for definitive chemoradiation, followed by intra-cavitary brachytherapy (ICBT).

**Figure 2:**
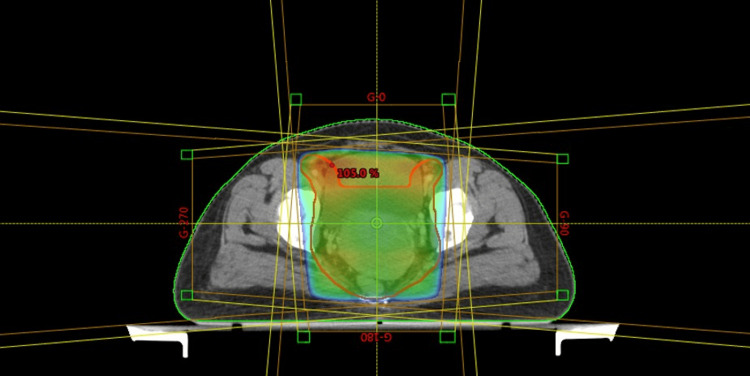
Phase 1 conformal EBRT plan for 50 Gy/25 Fr. EBRT: external beam radiotherapy

**Figure 3:**
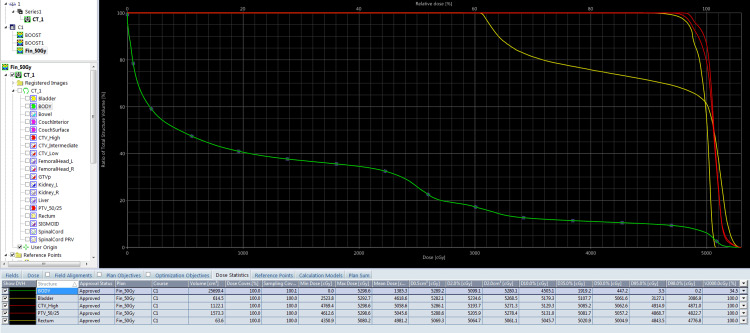
Dose-volume histogram of phase 1.

She was treated weekly with concurrent 50 mg doses of cisplatin and EBRT using conformal radiation therapy at a dose of 50 Gy/25 Fr with 2 Gy per fraction for five days a week as a phase 1 plan (Figure 2). Figure 3 shows the dose-volume histogram (DVH) for the EBRT treatment phase.

**Figure 4:**
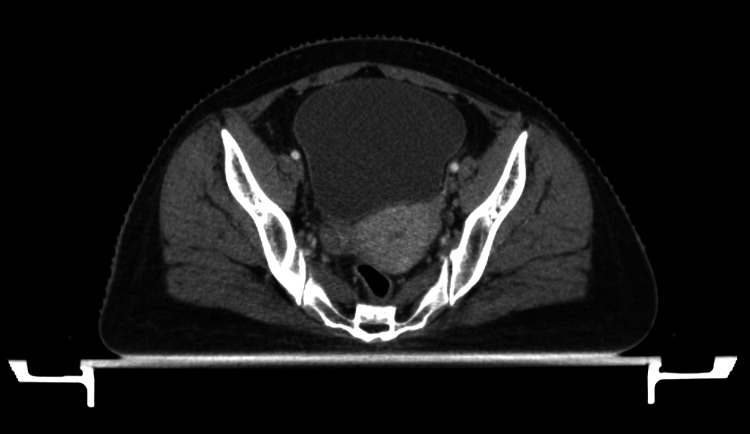
Response assessment planning CECT scan (axial section). CECT: contrast-enhanced computed tomography

**Figure 5:**
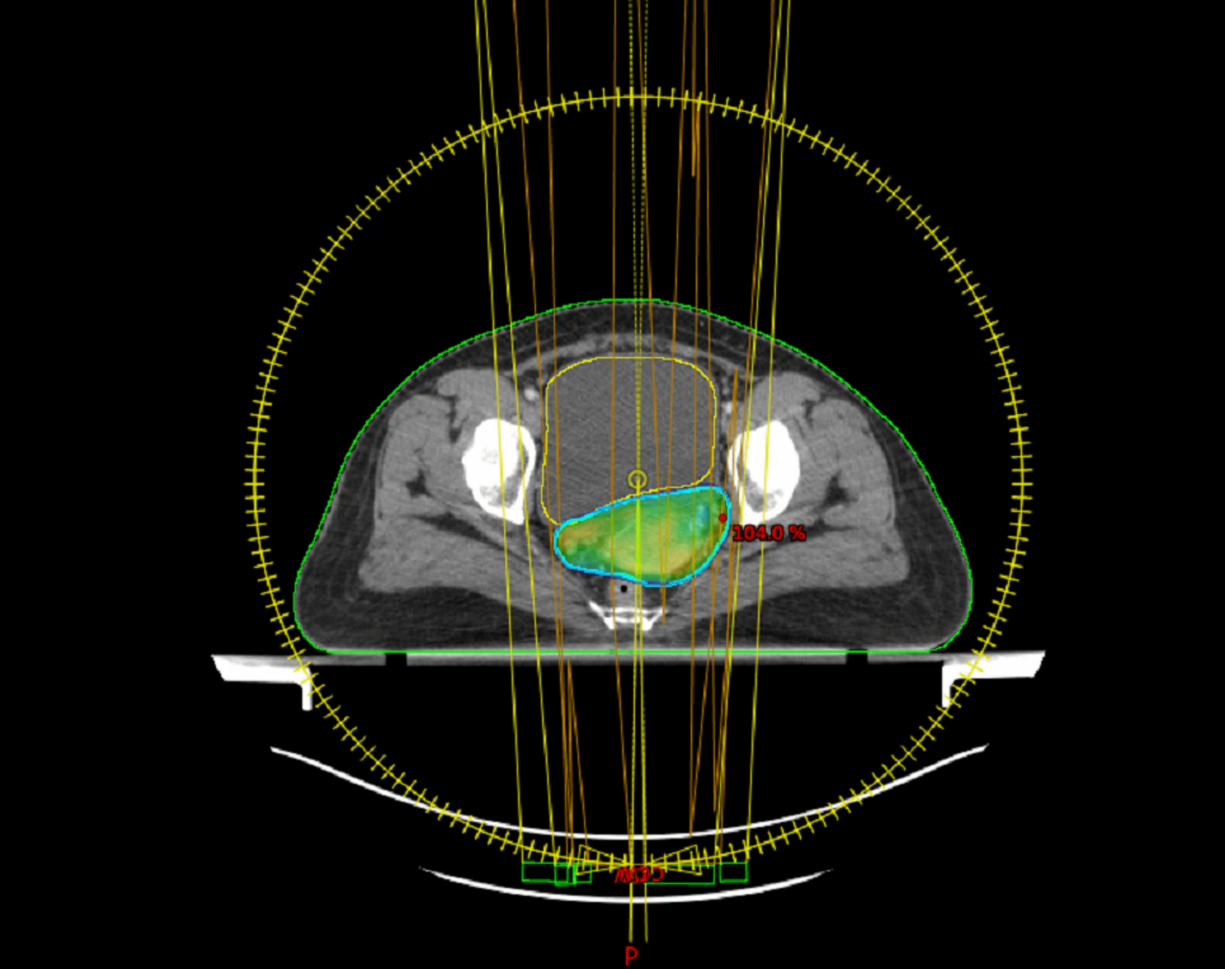
Phase 2 adapted VMAT boost plan for 8 Gy/4 Fr (axial section). VMAT: volumetric modulated arc therapy

**Figure 6:**
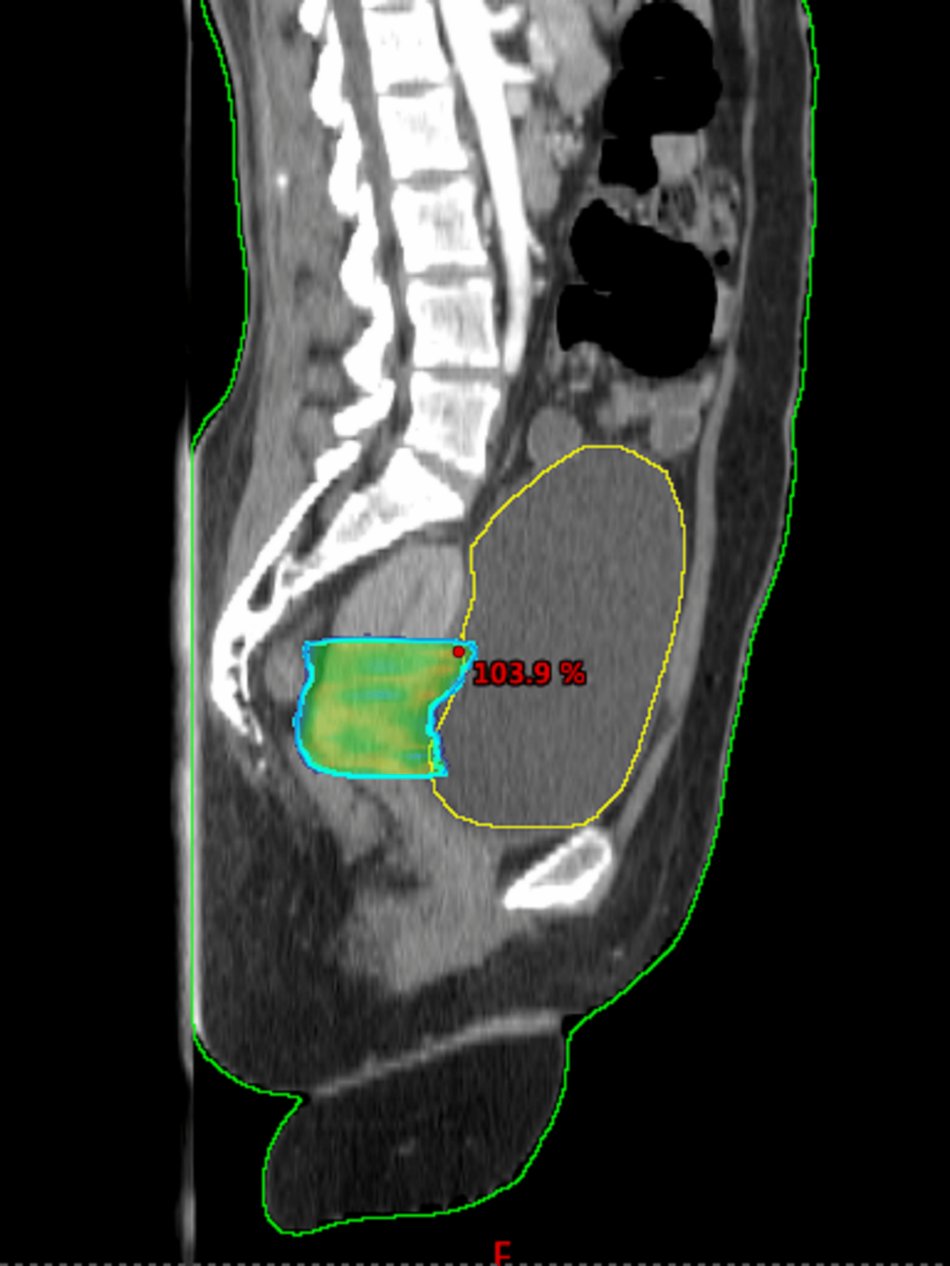
Phase 2 adapted VMAT boost plan for 8 Gy/4 Fr (sagittal section). VMAT: volumetric modulated arc therapy

**Figure 7:**
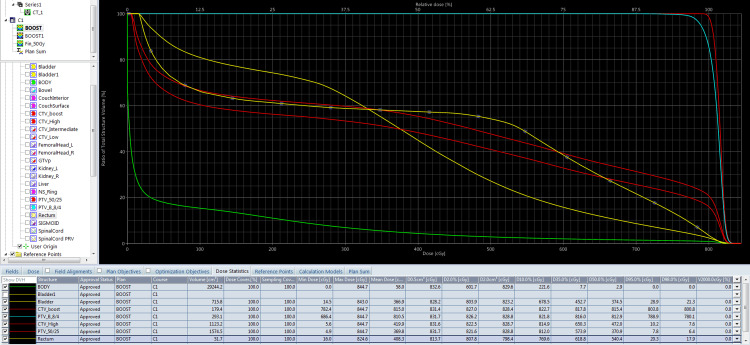
Dose-volume histogram of phase 2 (adapted boost plan).

Following a poor tumor response to the treatment as seen on the kvCBCT and a response assessment planning CECT scan (Figure 4), the patient was planned for an EBRT boost with VMAT of 8 Gy/4 Fr with 2 Gy per fraction as an adaptive radiotherapy protocol (Figures 5, 6). Combined (initial plan with boost plan), the maximum dose received by the bladder was 61.41 Gy, while the rectum received 59.05 Gy. Figure 7 presents the patient's EBRT DVH for the boost radiotherapy plan.

According to institutional protocol, the patient received weekly ICBT with 9 Gy/session for two sessions.

**Figure 8:**
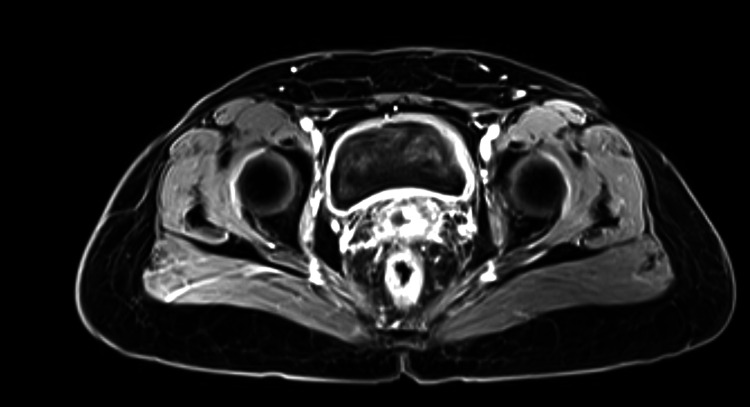
Follow-up CEMRI scan of the whole abdomen showing a complete response (axial section). CEMRI: contrast-enhanced magnetic resonance imaging

After four months, the contrast-enhanced MRI (CEMRI) scan of the whole abdomen indicated a complete response, and she proceeded with follow-up care. Subsequently, six monthly follow-up CEMRI abdominal scans until May 24 suggested a complete response (Figure 8). No significant long-term toxicity related to bladder and bowel was observed.

## Discussion

Radiation therapy techniques that enable modifications based on images acquired during treatment are referred to as adaptive radiotherapy techniques (ART). Modern imaging methods such as CT, MRI, and PET can be used by ART to adjust the treatment plan based on changes in anatomy and even biology that are noticed during the course of treatment [6-8]. Nowadays, ART is frequently employed as the standard of care for treating advanced head and neck carcinomas, ultra-central lung carcinomas, and other remotely located cancers. With the introduction of on-board imaging (OBI), ART is nowadays the standard of care in precision radiation therapy. The incidence of radiation-induced toxicity, such as xerostomia, proctitis, and cystitis, has decreased significantly with the use of ART. Adaptive radiotherapy planning approaches are often needed in cases of gynecological malignancy, particularly in cases where the tumors are still intact, as in cervical cancer cases, and more often in postoperative cases to match the bladder and rectal volume. Radiation therapy accuracy using adaptive IMRT is highly dependent on the target's motion and deformation and daily variations in the bladder and rectum.

Different approaches to ART are available, including customized margins based on an internal target volume (ITV) and offline and online approaches. Individualized ITV is one method of reducing the large population-based margins required to ensure adequate coverage of the CTV. It is generally used in gynecological, gastrointestinal, and thoracic malignancies. With the help of the ITV margin, there is a reduction in the chance of missing the target.

Offline adaptive strategies covered in the EBRT course include scheduled replanning and, more often, ad hoc replanning. It helps in tracking the inter-fraction changes of tumors and OARS.

Tumor volume variations and daily anatomical variations in target volumes and OAR are considered in the online ART (oART) strategy. In this approach, even intra-fraction changes can be assessed. Thus, PTV margins should be adequate enough to cover the target. A CBCT-guided oART system has recently been developed, although the majority of oART systems that are currently on the market are MRI-guided [9,10].

In the present case, an offline ART approach is used according to the institutional setup. Most of the centers are currently using ITV and offline-based ART. The present case shows an excellent amalgamation of conformal radiotherapy with adaptive VMAT as a boost, followed by ICBT. In general, EBRT boost is used to irradiate parametrium, and if at all used for residual tumor as a boost plan, then brachytherapy is seldom used. In this case, we used all of the above modalities to achieve a complete response to the tumor.

The limitation of this case report is that upfront IMRT- or VMAT-based treatment was not done as per institutional protocol, which dictates that IMRT should be used in postoperative cervix or endometrium cases and in node-positive cervical carcinoma cases. This caused an increase in the bladder and bowel dose. Fortunately, our case did not show any long-term toxicity related to the bladder and bowel, even after almost two years of treatment.

## Conclusions

From our case report, it is clear that one can achieve good dosimetric coverage and response even in tumors that are showing poor response to ongoing radiation treatment by assessing their nature based on changes in size and shape on a daily assessment with kvCBCT scans. With the help of such adaptive radiation therapy, one can save the adjacent critical OAR from acute as well as chronic toxicity. This case also shows that cancer treatment sometimes requires an all-around amicable approach between modern techniques, such as adaptive IMRT, and conventional ones, such as brachytherapy.

Adaptive radiotherapy should be used routinely for intra-fraction and inter-fraction assessment of tumor volume. It contributes to a higher therapeutic ratio for tumor control while also lowering the toxicity of the OAR. This should be the standard of care for all such cancers, which are deep-seated in nature and surrounded by critical organs.
